# Supplementary material for: Molecular Composition of Serum Exosomes Could Discriminate Rectal Cancer Patients with Different Responses to Neoadjuvant Radiotherapy
Source: Cancers (Basel). 2022 Feb 16;14(4):993. doi: 10.3390/cancers14040993 (PMC8870712; doi:10.3390/cancers14040993)
Supplement: Supplementary file 1 [file cancers-14-00993-s001.zip › Supplementary.pdf]

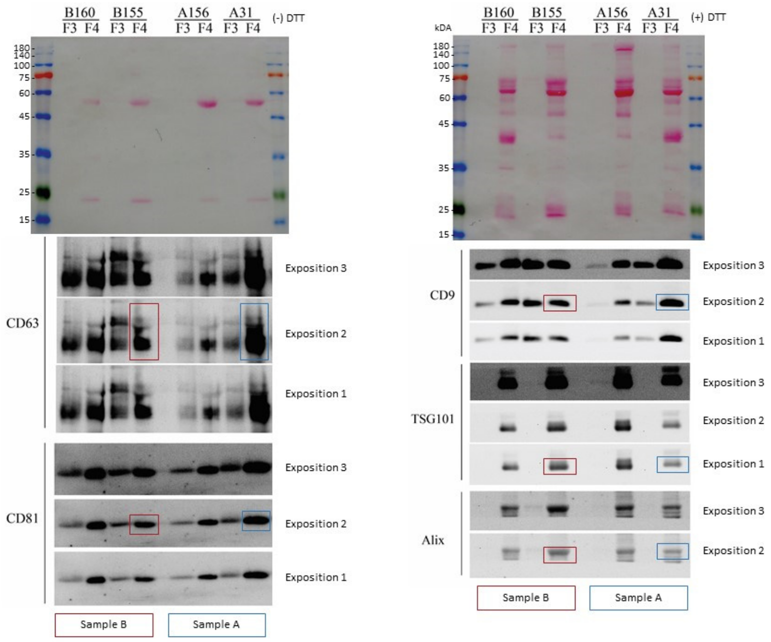

		total intensity
CD81	Sample A	79.51
	Sample B	55.87
CD63	Sample A	110.265
	Sample B	62.187
CD9	Sample A	95.72
	Sample B	63.16
TSG101	Sample A	27.781
	Sample B	57.546
Alix	Sample A	22.596
	Sample B	38.241

Figure S1. The original Western blot figures for analysis of exosomal markers in samples representative for good and poor responders A and B, respectively; included densitometry readings (total intensity) of chosen blots presented on Figure 1 (marked with a rectangle).

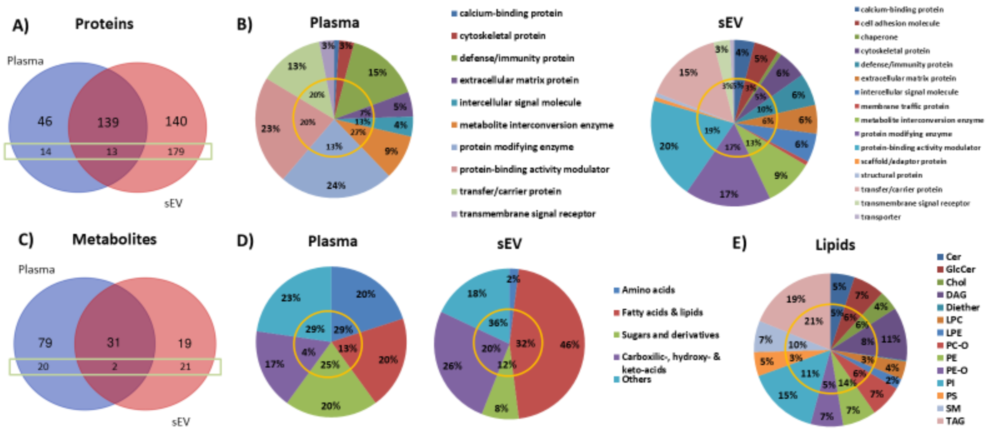

Figure S2 General characteristics of protein, metabolite, and lipid profiles in plasma and serum-derived exosomes of patients with rectal cancer. Panel A—Numbers of proteins detected in plasma and exosomes (differentially expressed proteins, DEPs, are delineated in a rectangle). Panel B—Contribution of detected proteins to different classes (DEPs are delineated in the internal cir-cle). Panel C—Numbers of metabolites detected in plasma and exosomes (differentially accu-mulated metabolites, DAMs, are delineated in a rectangle). Panel D—Contribution of detected metabolites to different classes (DAMs are delineated in the internal circle). Panel E—Contribution of lipids detected in plasma to different classes (differentially accumulated lipids, DALs, are delineated in the internal circle)

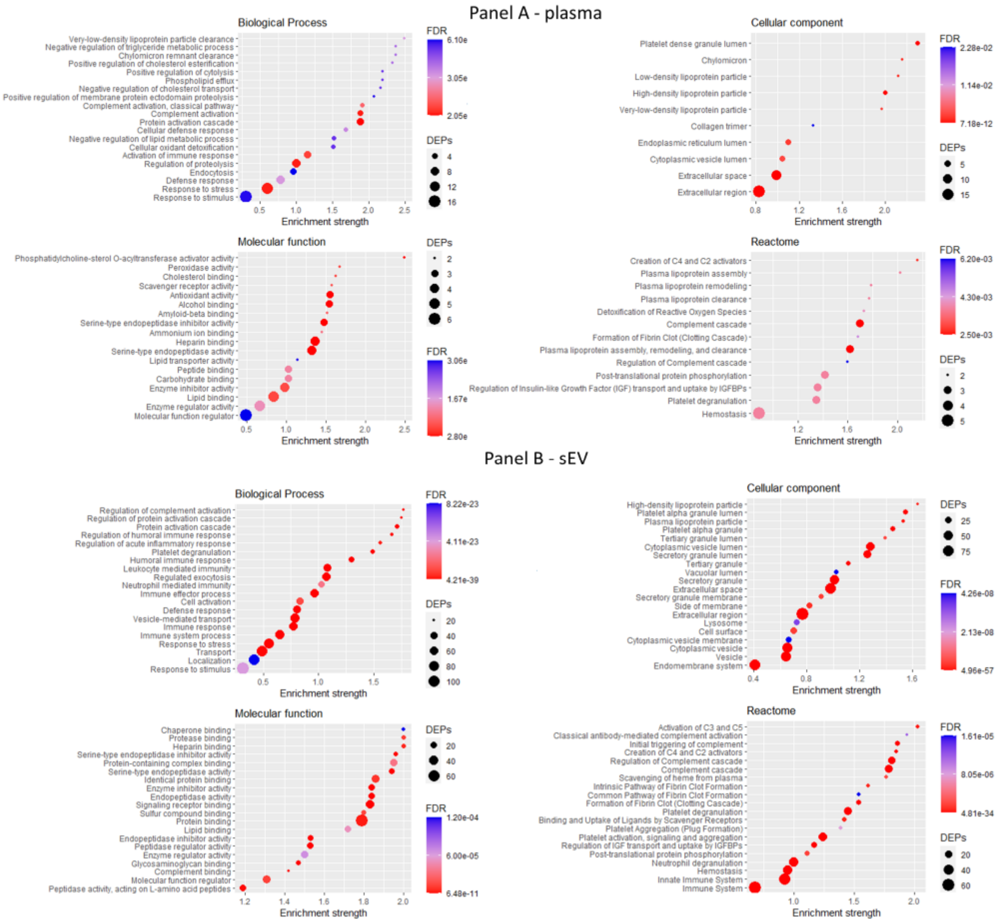

Figure S3. Functional enrichment analysis of differentially expressed proteins (DEPs) detected in plasma (Panel A) and exosome (Panel B) samples of patients with different responses to the treatment. Bubble plots of the TOP20 enriched biological process, molecular function, cellular component (based on GOterm), and Reactome pathways. The terms with the largest enrichment significance are plotted in order of enrichment effect (strength). The size of the dots represents the number of DEPs associated with the GO term/Reactome pathways and the color of the dots represents the p-adjusted values (Benjamini–Hochberg correction)

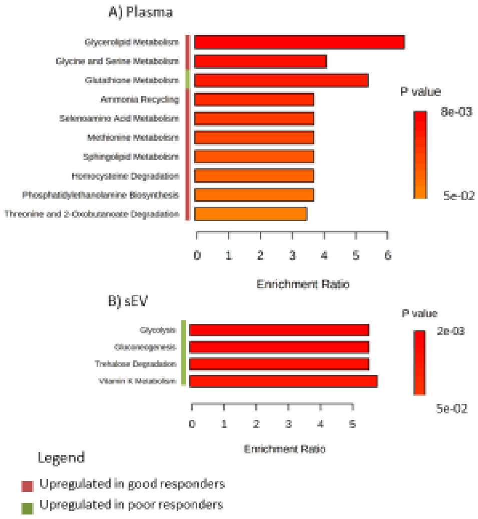

Figure S4. Metabolomic pathways associated with differentially accumulated metabolites (DAMs) detected in plasma (Panel A) and exosome (Panel B) samples. Presented are statistically significant over-represented pathways estimated by the quantitative enrichment analysis (QEA) algorithm.

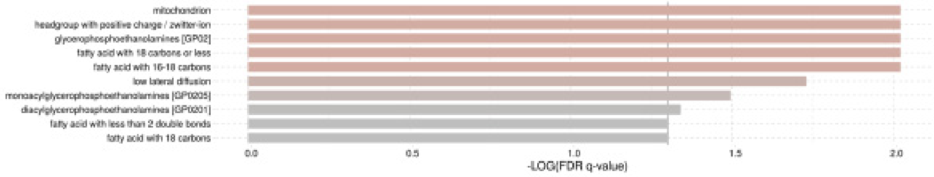

Figure S5. LION-term enrichment analysis of plasma lipids presented in the “ranking mode” as the differ-ence between good responders and poor responders. The gray vertical lines indicate the thresh-old of –log10 False discovery rate (FDR)-corrected p-value 0.05 (q-value 1.3). Red bars present terms with FDR-corrected p-value <0.01 (q >2).

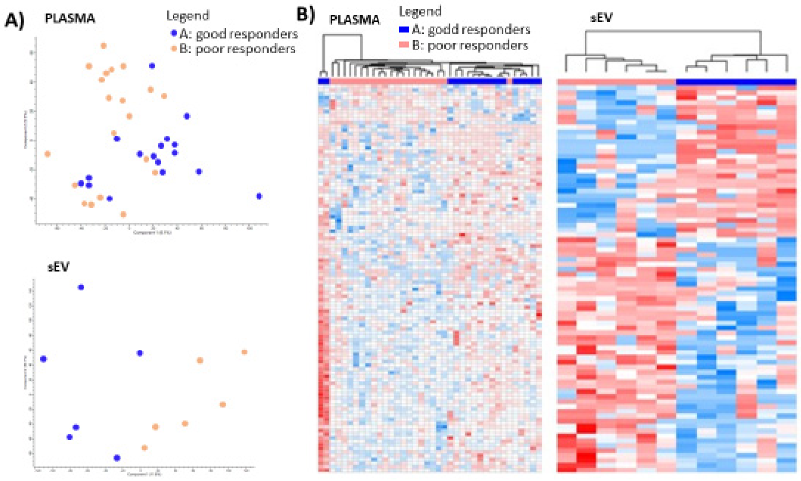

FigureS6**.** Unsupervised clustering of patients with rectal cancer based on levels of metabolites in plasma (Panel A) and exosome (panel B) samples. Showed are PCA score plots (first two components) and dendrograms resulting from the hierarchical cluster analysis; samples of good responders and poor responders are marked in navy blue and pink color, respectively.

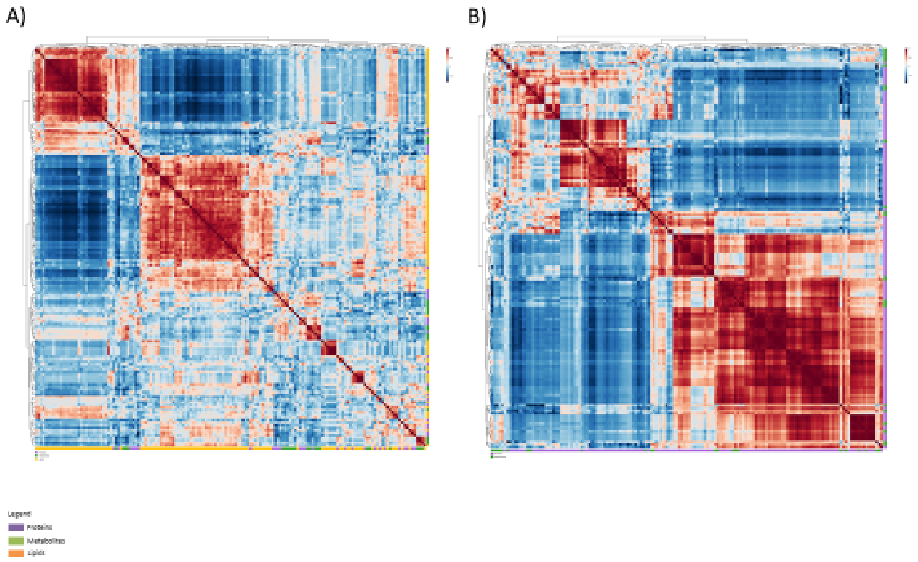

FigureS7**.** The correlation analysis was performed for differentially expressed proteins, metabolites, and li-pids detected in plasma (Panel A) as well as proteins and metabolites detected in exosomes (Panel B). Results are presented as a heat map with the corresponding hierarchical tree; color saturation corresponds to the value of Pearson's correlation coefficient (names of molecules were replaced by colors corresponding to the component class).

Supplementary methods

A. Protein extraction and digestion

Serum derived sEV were concentrated using Vivaspin 500 ultrafiltration tubes (100,000 MWCO, Sartorius, Göttingen, Germany) and 20μL of the concentrate (corresponding to 15–20μg of proteins) was mixed with 5μL of 0.5% sodium deoxycholate (SDC). In the case of plasma samples, 2 μL of plasma was diluted with 118μL of water, and next 10 μL of diluted plasma was transferred to new tubes and mixed with 100 μL of 1% sodium deoxycholate (SDC) in 50mM NH4HCO3 buffer. Then, plasma and serum derived sEV in SDC were homogenized using Precellys 24 homogenizer (Bertin Technologies, France) and sonicated in a bath for 10 minutes on ice. Samples were centrifuged for 10 minutes at 11,000 x g at 4°C and the supernatant was transferred to new tubes. Isolated protein extract was quantified using Pierce BCA protein assay kit (Thermo Scientific, Rockford, lL, USA) according to instructions delivered with the product. For in-solution digestion, ten-microliters of sample containing 10 μg of proteins were diluted with 15 μl of 50 mM NH4HCO3 buffer and then reduction with DTT (final conc. 5.6 mM) for 5 min at 95°C was performed. Next, thiol groups in proteins were alkylated using 5 mM iodoacetamide (IAA) for 20 min in the dark at room temperature. Digestion was done with 0.2 μg of sequencing-grade trypsin (Promega) per sample overnight at 37°C, then 1.5 μg of 10% trifluoroacetic acid (TFA) was added, mixed for 10 minutes and twice centrifuged for 7 min at 11,000 x g at room 20°C. The supernatant containing purified tryptic peptides was subjected to LC–MS/MS analysis.

B. Mass spectrometry analysis of proteins

The analyses were performed using a Dionex UltiMate 3000 RSLC nanoLC system coupled to a QExactive Orbitrap mass spectrometer (Thermo Fisher Scientific). The obtained protein digests were separated on an Acclaim PepMap RSLC nanoViper C18 reverse phase column (75 μm x 25 cm, 2 μm granulation) using an acetonitrile gradient (4 to 60%, in 0.1% formic acid) at 30°C and a flow rate of 300 nl/min (for 190 min). The spectrometer was operating in data-dependent MS/MS mode with survey scans acquired with a resolution of 70,000 at m/z 200 in MS mode and 17,500 at m/z 200 in MS2 mode. Spectra were recorded in the 300–2000 m/z scan range in the positive ion mode. Ion fragmentation with higher energy collision dissociation (HCD) was achieved with the normalized collision energies set to 25. All the raw data obtained for each dataset were imported into Protein Discoverer 2.1 package (Thermo Fisher Scientific) for protein identification and quantification. Protein identification was performed using the Swiss-Prot human database with a tolerance accuracy of 10 ppm for peptide masses and 0.08 Da for fragment ion masses. Two misscleavages per peptide were allowed and methionine oxidation as dynamic modification and carbamidomethylation of cysteines as static modification were set for all searches. Protein was considered as identified if at least 2 peptides per protein were found by the search engine, and a peptide score reached the significance threshold FDR = 0.01 (assessed by the Percolator algorithm). The abundance of identified proteins was normalization to the total ion current (TIC).

C. Metabolite extraction and derivatization

25 ul aliquots of plasma samples were deproteinized with 4 volumes of cold methanol and stored in freezer for 20 minutes at -20°C. Then, samples were centrifuged at 11,000 x g for 5 min at 4°C and supernatants were transferred to another Eppendorf tubes. Samples were then dried in vacuum centrifuge (CentriVap Concentrator, Labconco, USA) until derivatization. In the case of sEV, fraction

#4 was evaporated in vacuum centrifuge, then dried fraction was extracted subsequently with 200 ul of hexane, chloroform, methylene chloride and finally methanol. Each time after adding organic solvent, mixture was sonicated for 10 minutes, then centrifuged for 10 min at 11,000 x g at 4°C and next dried in vacuum centrifuge. The dried extract was then derivatized with 40 µl of methoxyamine hydrochloride in dry pyridine (20 mg/ml) at 37 °C for 1.5h with agitation. The second step of derivatization was performed by adding 90 µl of MSTFA (N-Trimethylsilyl-N-methyl trifluoroacetamide) and incubation at 37 °C for 30 min with agitation. Samples were subjected to GC/MS analysis directly after derivatization.

D. Mass spectrometry analysis of metabolites

Metabolites were separated and analyzed using the GC-MS system (TRACE 1310 GC oven with TSQ8000 triple quad MS from Thermo Scientific, USA) comprising a DB-5MS column (30 m × 0.25 mm × 0.25 µm) (J & W Scientific, Agilent Technologies, Palo Alto, California, USA). The conditions for the gradient during chromatographic separation were kept as follows: 70°C for 2 min, then 10°C/min to 300°C, at 300°C (10 min). The PTV injector was used to inject the sample with a temperature gradient from 40 to 250°C, the column interface was kept at 250°C, and the source temperature was set to 250°C. The EI ion source was operating in the m/z range of 50-850, the electron ionization energy was set at 70 eV. The retention index mixture containing alkanes was run prior to relevant analyzes. Raw data files were converted to abf format for analysis using MSDial software (v. 3.96). To eliminate the retention time (Rt) shift and to determine the retention indexes (RI) for each compound, the correction against alkane series mixture (C-10 to C-36) was implemented directly in MS Dial. For compound identification, MSP database from CompMS site containing 28,220 records was used. Metabolite was considered as identified if similarity index (SI) was above 80%. Identified artifacts (alkanes, column bleed, plasticizers, MSTFA, and reagents) were excluded from further analyses. Obtained normalized (using total ion current (TIC) approach) results were then exported to Excel for pre-formatting and then used for statistical analyses.

E. Lipid extraction

Lipid separation was carried out according to MTBE extraction protocol [31]. 20 µl of plasma were diluted with 80 µl of water and transferred to glass tubes with teflon-coated caps. Next, 750 µL of methanol and 2.5 mL of MTBE (tert-butyl methyl ether) were added and mixtures were vortexed for 1h at room temperature. Phase separation was induced by the addition of 625 µL of water. Upon 10 min incubation at room temperature, mixtures were centrifuged at 1,000 x g for 10 min and 2 mL of upper organic phase were then collected and dried under nitrogen stream at 37°C. Samples were directly resuspended prior to analysis in 300 µL of MS-mix buffer containing 7.5 mM ammonium acetate in chloroform, 2-propanol and methanol (1:2:4 v/v/v).

F. Mass spectrometry analysis of lipids

Lipid profiling of plasma samples was performed using Q-Exactive Orbitrap mass spectrometer (Thermo Fisher Scientific, Bremen, Germany) equipped with TriVersa NanoMate nanoflow ESI ion source (Advion BioSciences ltd., Ithaca, NY, USA). 10 µL sample aliquots were infused directly into mass spectrometer, after ion current stabilization, sample was measured for 10 min. The source was operated at gas pressure of 1.25 psi and ionization voltage was set to 1.05 kV. MS data were acquired in positive ion mode within the range of m/z 300 – 1500 at the resolution of 140,000 (at m/z 200, Full Width at Half Maximum, FWHM). Automatic gain control was set to target value of 3x106 and ion injection time (IT) was 100 ms. Raw MS data were converted into mzXML format and further

processed using LipidXplorer software (ver. 1.2.8.1) developed at Max Planck Institute of Cell Biology and Genetics in Dresden (Germany) [32]. Profiles were obtained by averaging 8 minutes of recorded mass spectra, from second minute to ninth minute within ten minutes of sample delivery time, while the first 60 seconds of sample injection were allowed for electrospray and analyte flow stabilization observed based on total ion current (TIC) variation. Subsequently, alignment was performed in order to match related peaks within the whole dataset. Defined signals in mass spectra for further processing and consideration were selected by setting the value of threshold to 1500. Particular lipid species were identified afterwards based on accurately determined masses related to mass accuracy better than 5 ppm. Intensities of each phospholipid peak were normalized to TIC.
